# Wood Esterification by Fatty Acids Using Trifluoroacetic Anhydride as an Impelling Agent and Its Application for the Synthesis of a New Bioplastic

**DOI:** 10.3390/ma16216830

**Published:** 2023-10-24

**Authors:** Prabu Satria Sejati, Firmin Obounou Akong, Frédéric Fradet, Philippe Gérardin

**Affiliations:** 1 LERMAB, Inrae, Université de Lorraine, 54000 Nancy, France; prabu-satria.sejati@univ-lorraine.fr (P.S.S.); firmin.obounou-akong@univ-lorraine.fr (F.O.A.); 2 Research Center for Biomass and Bioproducts, National Research and Innovation Agency (BRIN), Bogor 16911, Indonesia; 3 PLASTINNOV, IUT de Moselle-Est, Université de Lorraine, 57500 Saint-Avold, France; frederic.fradet@univ-lorraine.fr

**Keywords:** esterification, fatty acid, reactivity, TFAA, plastic, wood

## Abstract

Fatty acids (FA) and their derivatives with long alkyl chain structures are good candidates for wood esterification to confer thermoplastic properties to wood. Nevertheless, they do not react easily with hydroxyl groups of wood. In this study, we investigated the reactivity of wood with various fatty acids of different chain lengths using trifluoroacetic anhydride (TFAA) as the impelling agent in various reaction conditions. Generally, the esterification of fatty acids without solvents resulted in higher Weight Percentage Gain (WPG) and ester content than the reaction in the presence of CH_2_Cl_2_. The esterification reaction could be performed effectively at room temperature, though an increased reaction temperature provoked degradation of the esterified wood. WPG of 67% was obtained for the C3 and 253% for the C16 alkyl chain analogs, respectively. Nevertheless, the ester content was fairly uniform, with values between 10.60 and 11.81 mmol ester/gram of wood for all chain lengths. A higher quantity of reagent led to higher ester content, which tended to stabilize after a ratio of 1:4 wood and TFAA/FA. The esterification reaction was performed rapidly, with an ester content between 7.65 and 9.94 mmol ester/gram of wood being achieved only after 15 min of reaction. Fourier transform infrared spectroscopy (FTIR) analysis was performed to confirm the drastic chemical changes of wood before and after esterification. Morphological observation by scanning electron microscope (SEM), softening measurement by thermomechanical analysis (TMA), and contact angle measurements demonstrated the possibility of esterified spruce wood being applied as a new bioplastic.

## 1. Introduction

Fatty acids and their derivatives with long alkyl chain structures are good candidates for wood esterification to improve the thermoplastic properties of wood. Early studies for converting lignocellulosic material into thermoplastic materials used fatty acid chlorides in nonaqueous solvents such as N_2_O_4_-dimethylformamide (DMF) and pyridine [1,2]. Fatty acid chloride esterification using various solvents (DMF, CHCl_3_, and methyl *tert*-butyl ether) has been specifically studied [3]. Thiebaud et al. introduced esterification of a high quantity of fatty acid chloride without solvent at high temperatures in a special reactor with a nitrogen bubbling system using sodium hydroxide solution to capture the hydrogen chloride byproduct [4,5]. The same procedure was later adopted by Wu et al. [6].

The utilization of fatty acid chlorides was also performed in non-derivatizing solvents to obtain thermoplastic lignocellulosic materials. Early works on such systems focused on cellulose, using N,N-dimethylacetamide/lithium chloride (DMAc/LiCl) as the solvent system and N,N-dimethyl 1-4-aminopyridine (DMAP) as the catalyst. These cellulose solutions were then acylated with several fatty acyl chlorides from butyryl chloride (C4) to stearyl chloride (C18) [7,8,9]. Another class of non-derivatizing solvents, considered green or environmentally friendly solvents, are ionic liquids (ILs), which destroy the hydrogen bond network in the crystalline region of cellulose. Wood meal was completely dissolved in 1-butyl-3-methylimidazolium chloride ([C4mim]Cl) before esterification with octanoyl chloride [10]. Thermoplastic cellulose could be obtained in the ionic liquid 1-allyl-3-methylimidazolium chloride (AmimCl) with acyl chlorides [11]. However, ILs are very expensive, thus limiting their wide use in industrial applications.

Acyl chloride, a fatty acid derivative, is widely used in wood thermoplasticization due to its reactivity in wood esterification, though hydrogen chloride is released as a byproduct in the reaction. Fatty acids, on the other hand, do not react easily with the hydroxyl groups of wood. Arni et al. introduced the utilization of trifluoroacetic anhydride (TFAA) as an impelling agent to esterify dicarboxylic acids in wood, using benzene as a solvent to improve wood hydrophobicity [12]. The work was continued by Shiraishi et al. [13] and Nakano [14], using fatty acids as esterifying agents and TFAA in benzene to obtain thermoplastic materials.

The development of thermoplastic materials from the various components of lignocellulose biomass still represents challenging work [15]. Examples have included molded thermoplastic materials obtained through the extrusion of eucalypt wood with a high degree of acetylation [16] or through the injection of kneaded mulberry branches in NaOH/urea solution plasticized using glycerol and glycerol triacetate [17]. The extrusion or reactive extrusion of aspen pulp functionalized with benzethonium chloride (hyamine) and sulfuric acid in the presence of acetic anhydride or butyric anhydride has been reported as a way to achieve moldable and flowable thermoplastic materials [18,19].

Most of the procedures discussed above have used large amounts of reagents at relatively high temperatures, which restricts their widespread use in industrial applications. Our previous study successfully converted wood into thermoplastic materials using myristic acid as an esterifying agent and TFAA as a promoter [20]. The influence of fatty acid chain lengths on esterified sample properties was also investigated [21]. It is important to study the reactivity of several fatty acids of differing chain lengths under various reaction conditions to provide an overview of the optimum and efficient conditions that may, ultimately, be used in industry. In this study, we investigated the reactivity of wood with various fatty acids of different chain lengths using TFAA as the impelling agent in various reaction conditions and discovered possible applications as a new bioplastic alternative.

## 2. Materials and Methods

### 2.1. Sample Preparation

Spruce wood sawdust (*Picea abies*), sieved to 18 mesh, was extracted using a mixture of toluene/ethanol (1/2, *v*/*v*) for 4 h, followed by ethanol for 4 h using Soxhlet, and then dried at 103 °C for 24 h. Fatty acids selected for this work were propionic acid (C3, 99% purity), capric acid (C10, 99% purity), lauric acid (C12, 98% purity), and palmitic acid (C16, 98% purity). All the fatty acids were purchased from Alfa Aesar (Kandel, Germany) and Sigma-Aldrich (Steinheim, Germany), whilst TFAA (purity ≥ 99%) was purchased from Sigma-Aldrich. They were used without further purification.

### 2.2. Wood Esterification

Solutions of equimolar proportions of fatty acids and TFAA were combined for 30 min. to form mixed anhydride. Various ratios of oven-dried wood mass (m0) were then added to the mixed anhydride solution (Table 1) at room temperature and 50 and 100 °C in a closed tube for a duration between 15 min. and 24 h. without solvent and using CH_2_Cl_2_ as solvent. Esterified samples were successively washed with ethanol and water, followed by Soxhlet extraction with a mixture of ethanol and water (2/1, *v*/*v*) for 24 h. The different ratio variations of wood and reagent and different reaction durations allowed the reactivity of esterification to be studied. As a result of these variations, a treatment of 1:4 mass ratio of wood and TFAA and 4 h of reaction was selected for further characterization. The sample was then dried at 103 °C for 24 h to obtain the final mass (m1). The weight percent gain (WPG) was calculated as follows using Equation (1) [21]:

(1) $$\mathrm{WPG}\;\left(\%\right)=\frac{m1-m0}{m0}\times 100$$

The ester content (in mmol of ester per gram of wood) was calculated from the mass of reactant grafted on the wood per gram of wood divided by the molecular mass of fatty acid used for esterification (M) minus 18, which corresponded to the molecular mass of water released during esterification, using Equation (2) [21]:

(2) $$\mathrm{Ester}\;\mathrm{content}\;\left(\mathrm{mmol}\;\mathrm{ester}\;/\;g\;\mathrm{of}\;\mathrm{wood}\right)=\frac{\left(\frac{m1-m0}{m0}\right)}{M-18}\times 1000$$

### 2.3. Film Formation of Esterified Wood

Esterified spruce was placed in a mold of dimension 2 × 3 × 0.05 − 0.1 cm between two parchment papers, then hot-pressed using a laboratory press (LabManual 300, Fontijne Presses B.V., Rotterdam, The Netherlands) at temperatures between 120 and 180 °C under 10 MPa of pressure for 10 min to obtain a transparent sheet or film.

### 2.4. Fourier Transform Infrared (FTIR) Spectroscopy Analysis

FTIR spectra were measured on an ATR Perkin Elmer Spectrum 2000 spectrometer (Perkin Elmer Ltd., Beaconsfield, UK) equipped with a diamond cell. The spectra of the non-modified and acylated material were measured in the range of 4000–650 cm^−1^ at a resolution of 4 cm^−1^ and then were baseline corrected and normalized by means of the dedicated Spectrum 10 software (Perkin Elmer Ltd., Beaconsfield, UK). 

### 2.5. Morphological Observations

The pressed esterified wood sample and a pressed non-modified wood sample were observed with a Hitachi Tabletop Microscope TM 3000, Tokyo, Japan (SEM). The whole examination was carried out at room temperature, and the micrographs of surfaces were produced at various magnifications. Prior to the measurements, the films were made conducting by metallization using Au/Pd 0.1 mm. 

### 2.6. Thermomechanical Analysis (TMA)

Thermomechanical analysis (TMA) of esterified wood was performed using a Mettler Toledo TMA SDTA 840 instrument (Mettler Toledo SAS, Viroflay, France). A pressed esterified wood film was compressed under a constant load of 0.1 N in a heated chamber ranging from 30 to 260 °C at 10 °C/min heating rate. As the temperature increased, the load moved and recorded the deformation. The maximum value of deformation was then considered to be the softening temperature. The results were analyzed using STARe software DB V14.00 (Mettler Toledo SAS, Viroflay, France).

### 2.7. Contact Angle Measurements

Contact angle measurements were performed to study the hydrophobicity of the pressed esterified wood surface using a goniometer (Kruss FM40 EasyDrop, Hamburg, Germany) equipped with Easy Drop software. A drop of water was placed on the surface of the film, and then the contact angle between the water drop surface and the baseline was measured every 3 s from 0 to 60 s. The left and right contact angle data were averaged based on each capturing time. This measurement was repeated three times.

## 3. Results and Discussion

### 3.1. Effect of Solvent and Temperature on Esterification Reactivity

**Figure 1:**
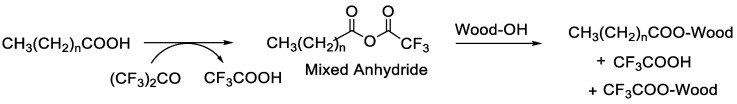
Chemical reaction of mixed anhydride formation and wood esterification [21].

Esterification of spruce wood occurred due to the reactivity of mixed anhydride of fatty acids and TFAA, allowing substitution of hydroxyl groups in wood by acyl chains as described in the reaction presented in Figure 1. The main product of this reaction was wood with an ester function comprised of a fatty acid chain and trifluoroacylated group, with volatile compounds as side products. Because of the complex and diverse structure of wood, the substitution of hydroxyl groups due to esterification was assessed by comparing the mass of the wood components before and after esterification and expressed as weight percent gain (WPG) and ester content.

**Figure 2:**
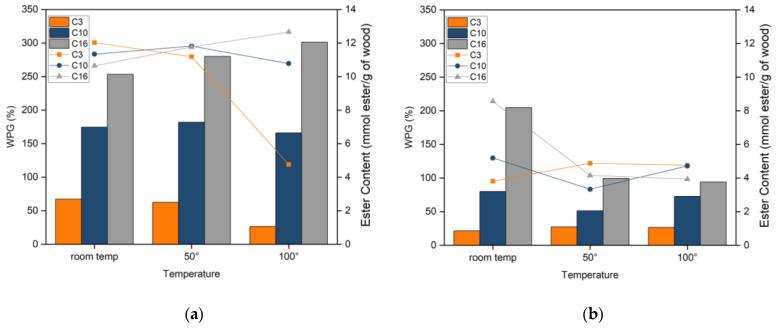
WPG and ester content of esterified wood at different temperatures without solvent (**a**) and in CH_2_Cl_2_ (**b**).

Figure 2 shows the relationship between WPG and the ester content of esterified spruce with different fatty acid lengths and reaction temperatures both in CH_2_Cl_2_ and without solvent. These three different fatty acid lengths were chosen as representative of short, middle, and long fatty alkyl chains to evaluate the effect of chain length on the reactivity and properties of the esterified wood. WPG differed depending on the number of carbon atoms in the fatty acid. As the length of the fatty chain increased, higher WPGs were obtained both in reactions without solvent and in CH_2_Cl_2_. Nevertheless, WPG does not always accurately represent the efficacy of esterification, especially when comparing different fatty acid lengths. For this reason, ester content was calculated by comparing WPG to the molecular weight of the fatty acid reacted. WPGs of esterified wood at room temperature without solvent were between 67 and 254%, and the ester content was fairly uniform, with values between 10 and 12 mmol ester/g of wood. Similar results regarding the uniformity of ester content were reported for fatty acid chlorides esterification wood and cellulose in various solvents [8,22]. However, different results were reported by Thibeaud et al. [4,5] for the solvent-free esterification with fatty acid chlorides and by Nakano [14] for the fatty acid esterification of wood, where it was suggested that lower WPGs were obtained when longer fatty chains were grafted to the wood, due to diffusion difficulties of high molecular weight fatty acids. This comparison indicates the benefit of using our method proposed of uniform accessibility for all fatty acid lengths to be esterified using the mixed anhydride method.

Wood esterification can be carried out at room temperature. Increasing the reaction temperature leads to a slightly higher WPG and ester content of long fatty acid solvent-free esterification. However, high reaction temperature decreases the WPG and ester content for esterification with short fatty acids. However, all esterified samples exhibited black and burnt aspects at 100 °C. Generally, higher WPGs and ester contents were achieved for esterification without solvent compared to treatment in CH_2_Cl_2_ for all fatty acids and temperatures of reaction. Direct contact between mixed anhydride and spruce wood without any solvents plays an important role during wood esterification, as shown by significantly higher ester content at room temperature solvent-free treatment than 100 °C treatment in CH_2_Cl_2_.

### 3.2. Effect of Duration and Reagent Quantity on Esterification Reactivity

**Figure 3:**
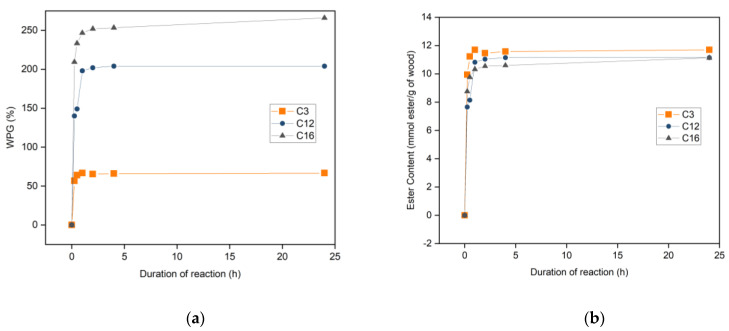
WPG (**a**) and ester content (**b**) of modified wood in different reaction durations.

The esterification rate of the selected fatty acids was investigated and presented in Figure 3. Propionic acid esterification of wood resulted in 56.7% WPG and 9.94 mmol ester/g of wood after 15 min of reaction, whilst lauric acid and palmitic acid esterification resulted in slightly lower ester contents of 7.65 and 8.76 mmol ester/g of wood, respectively. An ester content of 11.23 mmol ester/g of wood was achieved in propionic acid esterification after 30 min. Ester contents of about 10 mmol ester/g of wood were achieved in 1 h of reaction of lauric and palmitic acid esterification. The WPG and ester content started to stabilize at this time.

**Figure 4:**
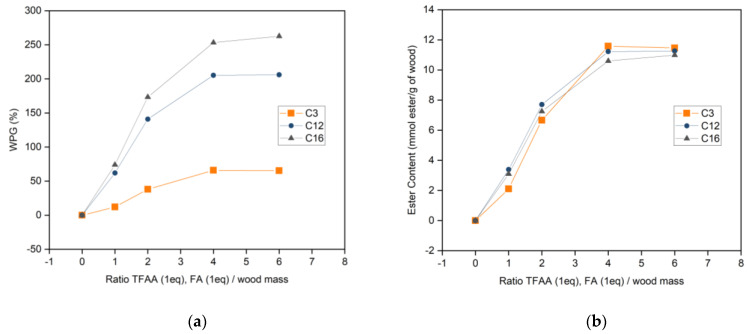
WPG (**a**) and ester content (**b**) of esterified wood in different quantities of reagent.

In order to better understand and optimize experimental conditions and the influence of each reagent used for the esterification of wood with fatty acids, experiments were performed using different ratios of the reagents. Figure 4 shows the relationship between the quantity of mixed anhydride to WPG and the ester content of esterified wood. A high quantity of mixed anhydride led to higher ester content when the ratio was changed from 1:1 to 1:4 wood to TFAA/FA. WPG and ester content tend to stabilize afterward at ester content comprised between 10.6 and 11.58 mmol ester/g of wood. This indicated the maximum capacity of hydroxyl groups available to be esterified was achieved at a ratio of wood and TFAA/FA of 1:4. There were no significant differences in ester content between the fatty acids used.

### 3.3. Chemical Structure Transformation of Esterified Wood

**Figure 5:**
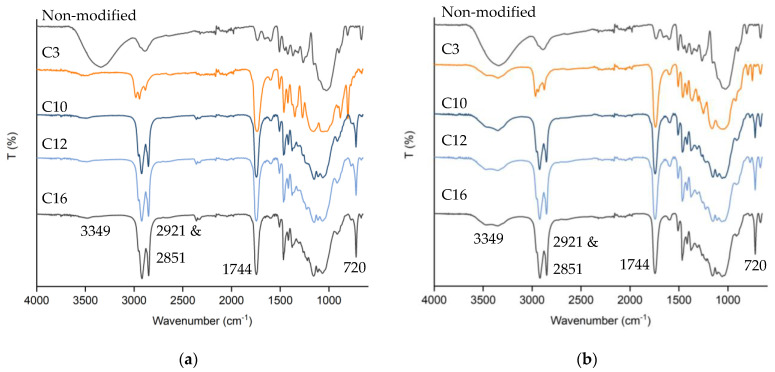
FTIR spectra of spruce wood before and after esterification with different fatty acids without solvent (**a**) and in CH_2_Cl_2_ (**b**).

Fourier transform infrared spectroscopy (FTIR) analysis was performed to establish the chemical structure transformation due to esterification. As presented in Figure 5, a significant decrease in the O-H stretching vibration band at 3349 cm^−1^ was observed for all lengths of fatty acid chain grafted to the esterified wood without solvent. However, the O-H stretching vibration peak was still observed in the esterified sample in the presence of CH_2_Cl_2_, indicating that more hydroxyl groups of wood were successfully esterified, confirming the higher WPG obtained without solvent. Successful esterification was confirmed with the appearance of three new peaks, first at 1744 cm^−1^, which corresponded to the adsorption of C=O carbonyl ester groups with relatively uniform intensity, confirming uniform ester content in each fatty acid used. Second peaks observed at 2921 and 2851 cm^−1^ corresponding to symmetric and asymmetric chains (–CH_2_–) [6,23,24,25,26] were present in different intensities corresponding to the length of fatty chains grafted. The third new peak at 720 cm^−1^ could be assigned to the characteristic of at least four linearly connected methylene (–CH_2_–) groups [8] in capric-, lauric-, and palmitic-acid-esterified wood.

### 3.4. Application of Esterified wood as New Bioplastic 

Esterified wood was transformed into plastic film using a thermocompression process.

**Figure 6:**
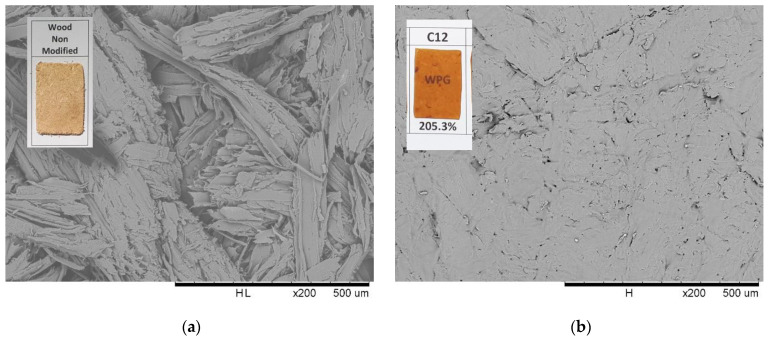
Visual appearance and scanning electron micrograph of pressed non-modified (**a**) and esterified spruce wood (**b**).

Surface properties and thermoplastic aspects were examined using SEM, TMA, and contact angle measurement to understand the possible application of esterified spruce wood as a new bioplastic. Without modification, pressed spruce wood resulted in a brownish opaque and woody textured surface, as observed in Figure 6a. A translucent yellowish film was obtained by pressing C12 esterified wood and confirmed by scanning micrograph in Figure 6b with the disappearance of a fibrous wood aspect, which changed to a smooth and homogenous surface, indicating self-integration and complete melting were achieved after hot pressing.

**Figure 7:**
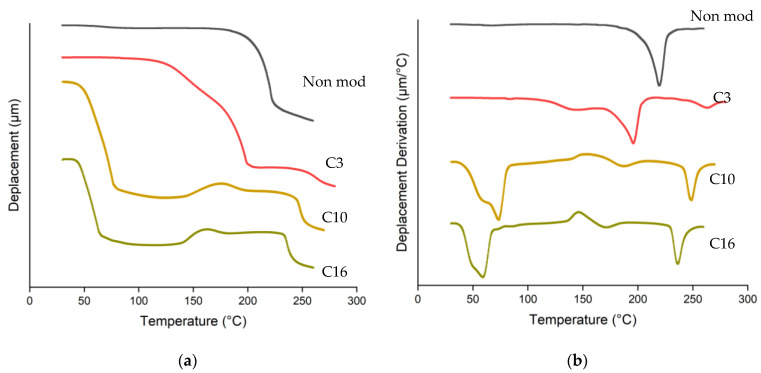
TMA (**a**) and DTMA (**b**) of spruce wood before and after esterification.

Thermoplastic properties of esterified spruce film were investigated using TMA by measuring the deformation of the film due to constant load in different temperatures (Figure 7). Before esterification, deformation of the non-modified spruce sheet was observed at 220 °C. After esterification, several deformations occurred at lower temperatures. As the length of the fatty chain increases, the first deformations were observed at 196, 72, and 56 °C for C3, C10, and C16, respectively. This softening temperature indicated the loss of film rigidity related to the fatty chain grafted to the wood [20,21]. The second deformation was observed between temperatures of 171 and 266 °C. Glass transition temperature observations were reported in the same range of temperature, corresponding to the reorganization of the fatty acid chain and cellulose backbone in oleic-acid-esterified cellulose [26]. The third deformation was observed between 245 and 257 °C for C16 and C10, respectively, which was associated with the onset of decomposition of the wood component [21].

**Figure 8:**
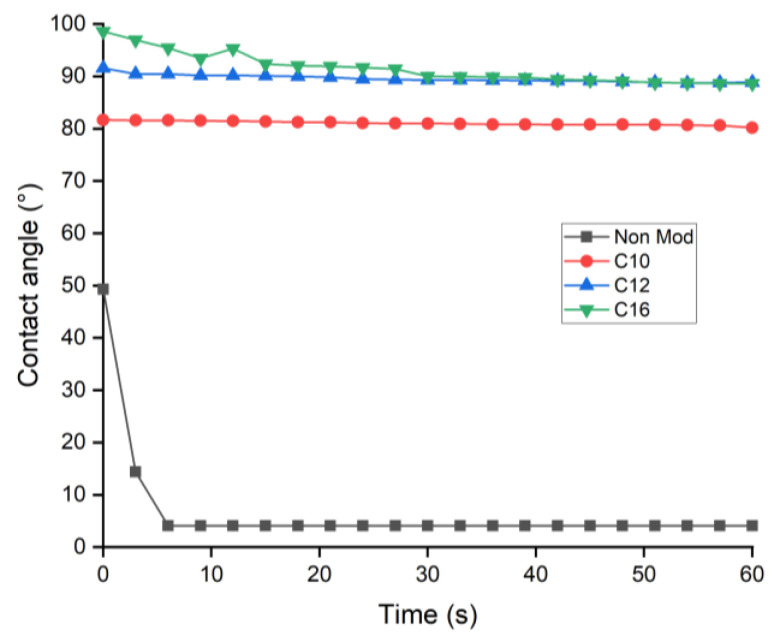
Contact angle improvement of esterified wood.

Figure 8 presents the hydrophobic aspect of esterified film by measuring the contact angle of the water droplet and pressed film surface. The low contact angle formed on the surface of the wood sheet before esterification shows a hydrophilic aspect, indicating the hygroscopic properties of native wood. Drastic improvement of the contact angle obtained in the esterified film was shown by the high and stable contact angle between 80 and 90° at the end of the measurement. Longer fatty acid chain esterification showed a slightly higher contact angle than shorter fatty acid analogs. The high contact angle obtained in esterified film demonstrated that fatty acid esterification of wood enhanced surface hydrophobicity and confirmed the elimination of free hydroxyl groups shown by FTIR data.

## 4. Conclusions

The reactivity of fatty acid esterification using TFAA as an impelling agent in various reaction conditions on spruce wood was investigated. WPG of esterified wood varied according to the length of fatty chains reacted but showed a uniform value of ester content, indicating uniform accessibility of all fatty acid lengths to be esterified using the proposed method. Direct contact between mixed anhydride and spruce wood plays an important role during wood esterification, as indicated by the higher WPG and ester content in treatment without solvent. Wood esterification could be carried out at room temperature; increasing temperature led to degradation and decreasing WPG of propionic-acid-esterified wood. Shorter fatty acids used in the reaction led to higher ester content achieved in a relatively faster duration. Increasing the quantity of reagent led to higher WPG and ester content, with maximum esterification of hydroxyl groups achieved at a ratio of 1:4 wood and TFAA/FA. Successful esterification was confirmed by FTIR results with a decrease in the O-H stretching vibration band simultaneously with the appearance of C=O carbonyl ester groups and aliphatic chain. The translucent film and the disappearance of the fibrous aspect observed by SEM, the low softening temperature observed by TMA, and the high contact angle demonstrate the possible application of esterified wood as a new bioplastic alternative.

## Figures and Tables

**Table 1:** Ratio variation of wood and TFAA and fatty acid solution (m/m for wood and TFAA).

Wood	TFAA	Fatty Acids
1	1	1
1	2	2
1	4	4
1	6	6

## Data Availability

Data are contained within the article.
